# Optimization of beam-flow angles for Doppler ultrasound flow velocity measurements using slanted gel pads

**DOI:** 10.1186/s40064-016-1977-z

**Published:** 2016-03-15

**Authors:** Michael Yong Park, Seung Eun Jung, Joon-Il Choi, Jae Young Byun

**Affiliations:** Department of Radiology, Seoul St. Mary’s Hospital, College of Medicine, The Catholic University of Korea, 222 Banpo-Daero, Seocho-gu, Seoul, 06591 Korea

**Keywords:** Pulsed Doppler ultrasonography, Ultrasound, Peak systolic velocity, Beam-flow angle, Angle correction, Spectral broadening

## Abstract

The aim of this study was to assess whether slanted gel pads can be used to optimize beam-flow angles and flow velocity measurements for Doppler ultrasound. The right carotid artery of a single healthy female volunteer was measured alternatively five times without and with an 18° angled slanted gel pad between the ultrasound transducer and skin by 13 radiologists. Beam-flow angles and peak systolic flow velocities (PSV) were measured along with assessment of spectral broadening. Beam-flow angles (*P* = 0.001) and PSV (*P* = 0.001) measurements showed a significant decrease when using slanted gel pads. The mean (±SD) beam-flow angles without and with the use of slanted gel pads were 66.7 (±4.2) and 56.1 (±5.8) degrees, respectively. The mean (±SD) PSVs without and with the use of slanted gel pads were 92.0 (±17.4) and 76.9 (±10.9) cm/s, respectively. There was a noticeable decrease in spectral broadening when using slanted gel pads. There was a significant linear correlation between beam-flow angle and peak systolic velocity. Coefficients of variation for peak systolic velocity without and with the use of gel pads were 18.9 and 14.2 %, respectively. These results demonstrate that slanted gel pads decrease beam-flow angles and overestimation of Doppler flow velocity measurements while potentially increasing the reliability of measurements.

## Background

Pulse Doppler velocity measurements are often used for the clinical evaluation of vascular stenosis, vascular patency, hepatic venoocclusive disease, and complications related to organ transplantation (Heijenbrok-Kal et al. 2006; Park et al. 2008). Although many textbooks recommend obtaining Doppler velocity measurements at less than 60°, prior published papers have suggested that there is no such clear cutoff value for accurate velocity measurements (Park et al. 2012; Hoskins 1996). Previous studies show that even with appropriate angle correction, increased beam-flow angles cause a significant overestimation of flow velocity, which is mostly due to spectral broadening (Park et al. 2012; Hoskins 1996). The reason for increases in overestimation of PSV as beam-flow angles increase is mostly due to intrinsic spectral broadening which shows marked increases as angles increase. Doppler frequency shifts are dependent on the cosine range of beam-flow angles. As the angles are increased the beam will be more slanted toward the Doppler aperture, which causes an increase in the small range of angles that the insonated point subtends to. This results in a larger range of Doppler frequency shifts, hence causing increased spectral broadening and overestimation of velocity (Park et al. 2012; Winkler and Wu 1995). Therefore, a similar beam-flow angle should be used between studies for more precise comparisons and a small beam-flow angle should be used for more accurate velocity measurements (Park et al. 2012).

When the flow of vessels are flowing nearly parallel to the body surface such as in the carotid arteries, portal veins, and femoral arteries, it is often difficult to obtain a small beam-flow angle during ultrasound studies. In such cases a large beam-flow angle is used during pulse Doppler velocity measurements which often leads to significant overestimation of flow velocity (Park et al. 2012; Hoskins 1999).

The purpose of this study is to evaluate the use of slanted gel pads between ultrasound probes and patients which can decrease beam-flow angles and subsequently decrease overestimation while potentially increasing the reliability of pulse Doppler flow measurements, especially in vessels flowing nearly parallel to the body surface.

## Methods

**Fig. 1:**
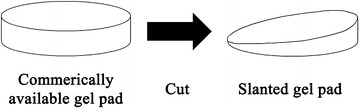
Diagram shows a commercially available gel pad which was cut diagonally to create an 18° slanted gel pad

**Fig. 2:**
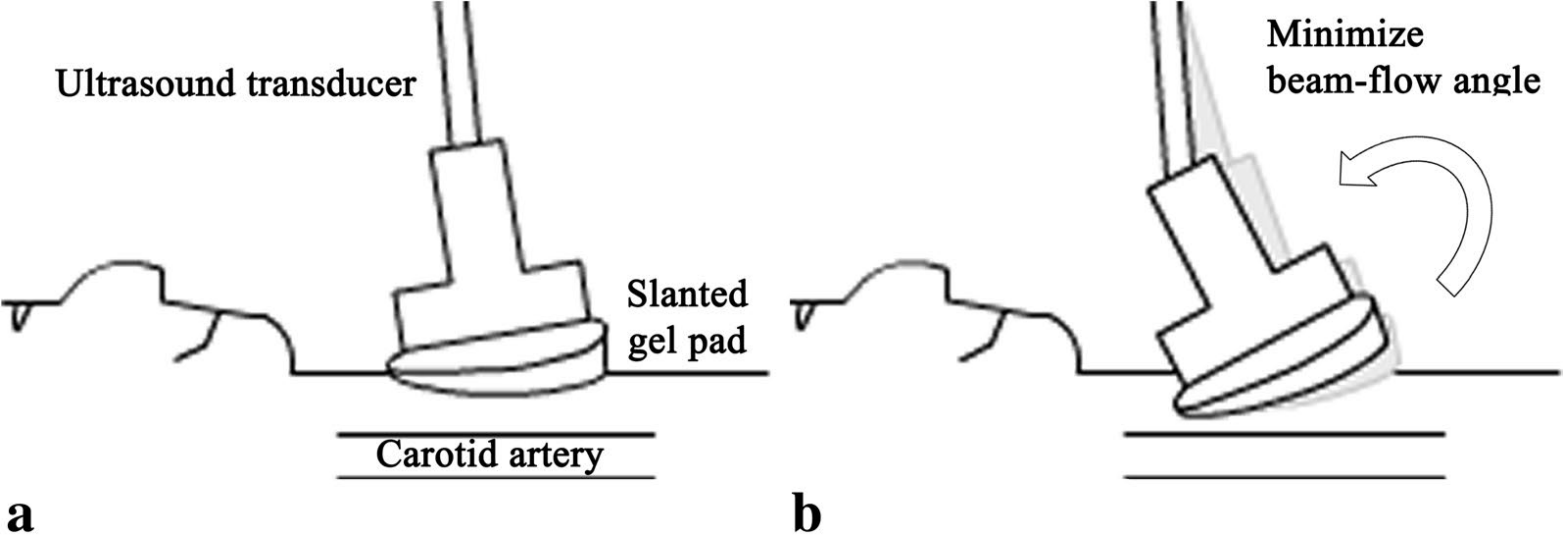
Diagram shows measurement of the carotid artery using a slanted gel pad to decrease beam-flow angle. **a** Diagram shows a slanted gel pad placed between the ultrasound transducer and skin which is decreasing the beam-flow angle. **b** As the skin and soft tissue is slightly elastic the probe and slanted gel pad can be tilted and compressed against the neck during measurement to further decrease beam-flow angle. This technique was left to the radiologists to perform according to personal preferences

This prospective study received institutional review board approval, and written informed consent to participate was obtained from a single healthy 31 year old, 162 cm height, 56 kg (body mass index: 21.3) female volunteer. An 18° angled slanted gel pad was made by diagonally cutting a commercially available ultrasound gel pad (2 cm × 9 cm, Aquaflex, Parker laboratories, Fairfield, NJ, USA) (Fig. 1). The right carotid artery of a single volunteer was measured alternatively 5 times without and with an 18° angled slanted gel pad between an ultrasound probe and skin by 13 radiologists (Fig. 2). Except for two radiologists who designed the study and slanted gel pads, the other radiologists were not aware of the purpose of this study and were told only that the effect of the slanted gel pad was to be evaluated. It was up to them to decrease beam-flow angles according to their usual personal ultrasound preferences with Doppler angle correction (Fig. 2b). The ultrasound experience of the participating 13 radiologists ranged between 3 to 19 years (mean 11.5 years). Beam-flow angle and peak systolic velocity (PSV) were recorded.

Pulse Doppler ultrasonography was performed with an iU22 (Philips healthcare Bothell, WA, USA) ultrasound unit using a high-frequency 7–12 MHz linear transducer. Velocity waveforms were obtained in the center stream approximately 2 cm below the bifurcation. A sample volume of 2.5 mm was used for PSV measurements.

Beam-flow angles and PSVs were each paired into two groups, one without the use and one with the use of slanted gel pads. The Wilcoxon signed rank test was used to check for significant differences between the two groups. Linear regression was used to compare the relationship of beam-flow angle and PSV with statistical significance determined by analysis of variance. Coefficients of variation for PSV without and with the use of slanted gel pads were used to compare reliability.

## Results

**Fig. 3:**
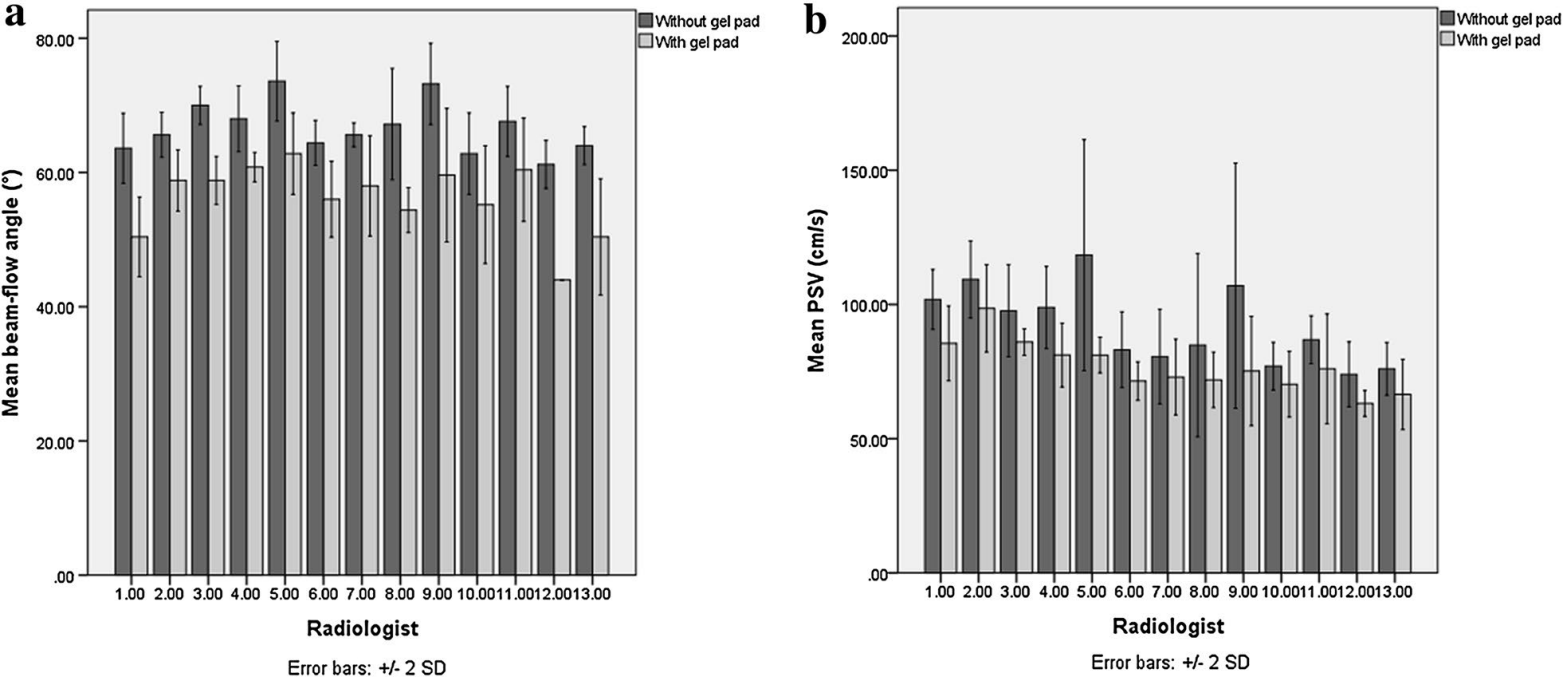
*Bar charts with error bars* showing the mean beam-flow angles and mean PSVs without and with the use of slanted gel pads by each radiologist. Radiologists 12 and 13 designed this study and were aware of the purpose of the study, the other radiologists were not aware of the purpose of this study. **a** *Side*-*by*-*side bar chart* showing beam-flow angles of each radiologist without and with gel pads. **b** *Side*-*by*-*side bar chart* showing the mean PSV of each radiologist without and with gel pads

**Fig. 4:**
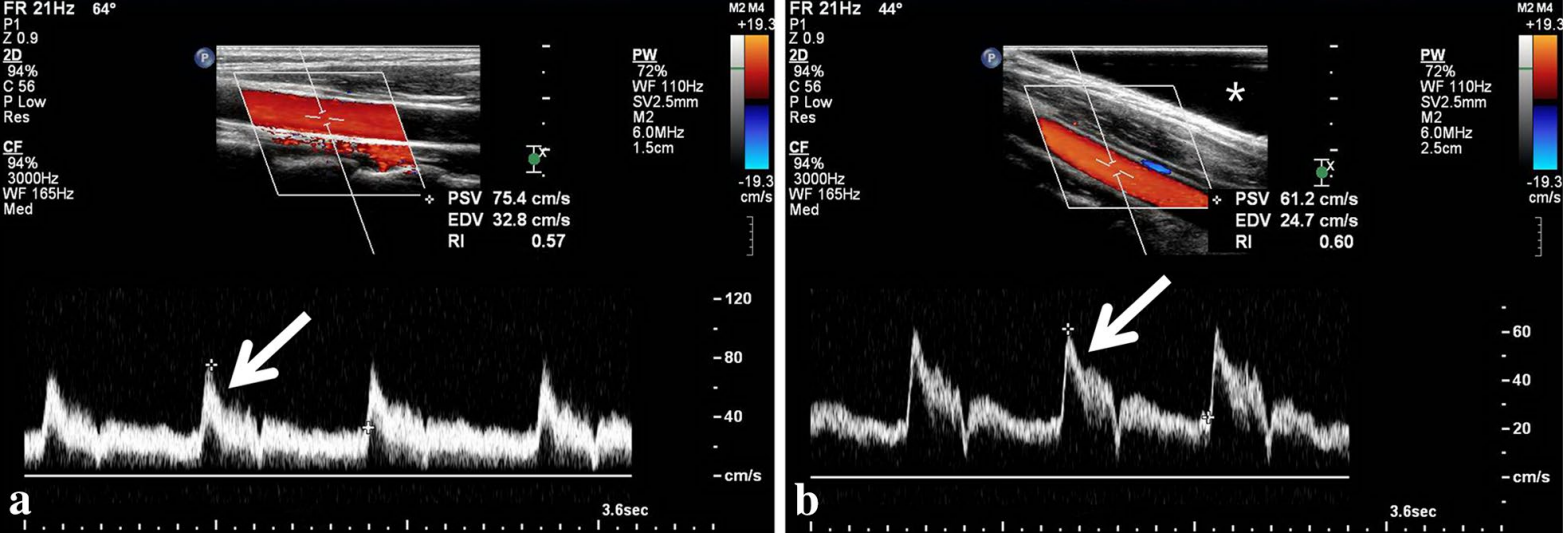
As Doppler beam-flow angles decreased, decreases in spectral broadening were seen. **a** Image of Doppler velocity measurement taken at a 64° beam-flow angle without the use of slanted gel pads shows spectral broadening throughout nearly the whole velocity range (*arrow*). **b** Image of Doppler velocity measurement taken at a 44° beam-flow angle with the use of a slanted gel pad (*star*) shows much decreased spectral broadening seen mostly throughout only the upper velocity range (*arrow*)

**Fig. 5:**
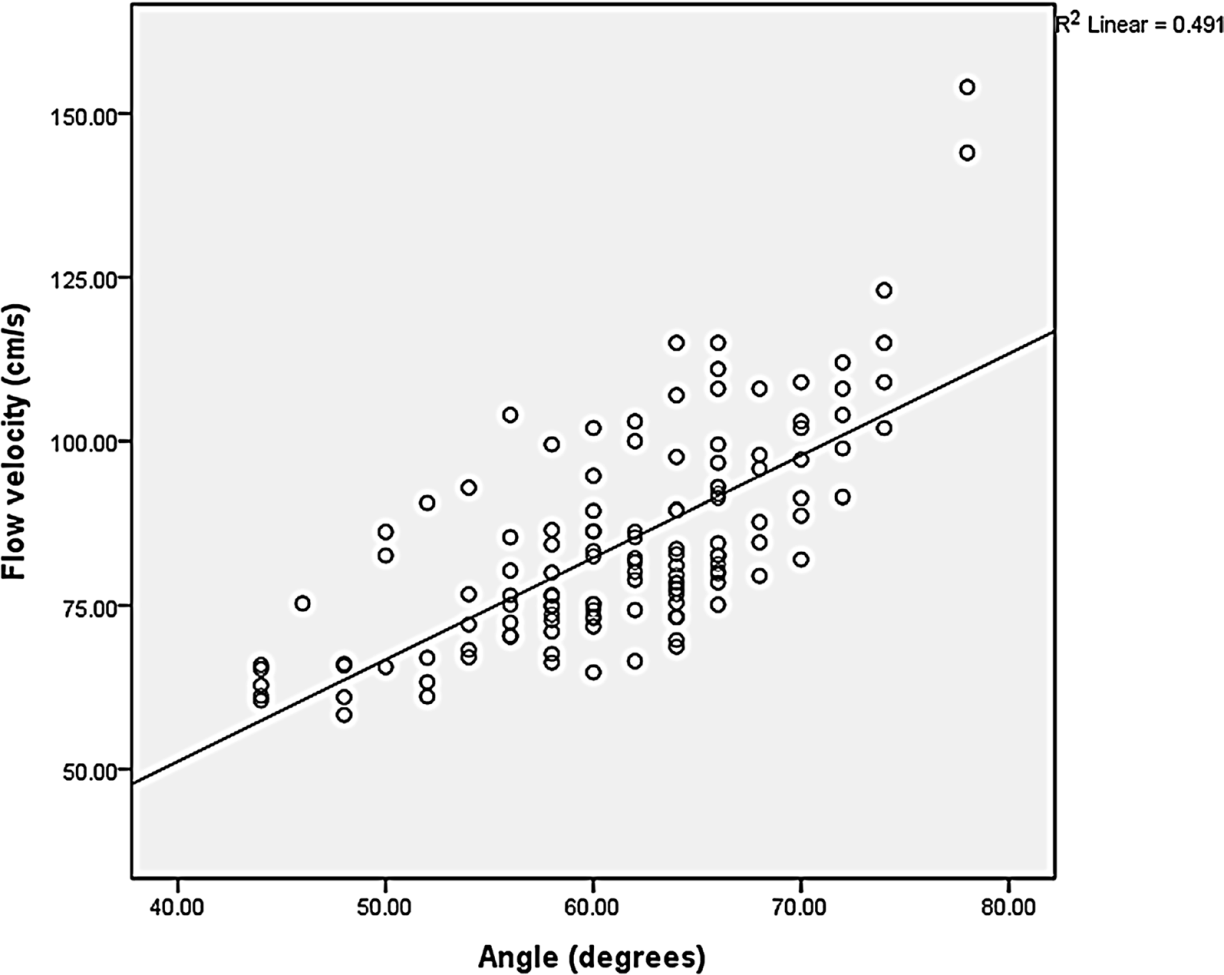
Scatter plot with line fit of flow velocity and angle shows a significant linear correlation (*P* = 0.000, R^2^ = 0.491) between beam-flow angle and PSV, with PSV overestimation progressively increased as beam-flow angles increased even with Doppler angle correction

Beam-flow angles (*P* = 0.001) and PSV (*P* = 0.001) measurements showed a significant decrease when using slanted gel pads (Table 1; Fig. 3), showing that overestimation of PSVs significantly decrease with the use of slanted gel pads. The mean (±SD) beam-flow angles without and with the use of slanted gel pads were 66.7 (±4.2) and 56.1 (±5.8) degrees, respectively. The mean (±SD) PSVs without and with the use of slanted gel pads were 92.0 (±17.4) and 76.9 (±10.9) cm/s, respectively. There was a noticeable decrease in spectral broadening when using slanted gel pads (Fig. 4). There was a significant linear correlation (*P* < 0.001, R^2^ = 0.491) between beam-flow angle and PSV, with PSV overestimation progressively increasing as beam-flow angles increased (Fig. 5). Coefficients of variation for PSV without and with the use of gel pads were 18.9 and 14.2 %, respectively.

**Table 1:** The mean (±SD) of beam-flow angles, PSVs without and with the use of slanted gel pads by radiologist and all radiologists combined. Radiologists 12 and 13 designed this study and were aware of the purpose of the study, the other radiologists were not aware of the purpose of this study

Radiologist	Mean beam-flow angle without gel pads (°)	Mean beam-flow angle with gel pads (°)	Mean PSV without gel pads (cm/s)	Mean PSV with gel pads (cm/s)
1	63.6 (±2.6)	50.4 (±3.0)	101.8 (±5.6)	85.5 (±7.0)
2	65.6 (±1.7)	58.8 (±2.3)	109.3 (±7.2)	98.6 (±8.1)
3	70.0 (±1.4)	58.8 (±1.8)	97.7 (±8.6)	86.0 (±2.5)
4	68.0 (±2.4)	60.8 (±1.1)	99.9 (±7.7)	81.1 (±6.0)
5	73.6 (±1.2)	62.8 (±4.0)	118.4 (±7.6)	81.1 (±7.9)
6	64.4 (±1.7)	56.0 (±2.8)	81.1 (±7.1)	71.5 (±3.4)
7	65.6 (±0.9)	58.0 (±3.7)	80.6 (±8.8)	73.0 (±7.1)
8	67.2 (±4.1)	54.4 (±1.7)	84.8 (±17.1)	71.9 (±5.2)
9	73.2 (±3.0)	59.6 (±5.0)	107.0 (±22.8)	75.2 (±10.2)
10	62.8 (±3.0)	55.2 (±4.4)	77.0 (±4.4)	70.3 (±6.1)
11	67.6 (±2.6)	60.4 (±3.9)	86.9 (±4.5)	76.0 (±10.3)
12	61.2 (±1.8)	44.0 (±0)	74.0 (±6.0)	63.1 (±2.4)
13	64.0 (±1.4)	50.4 (±4.3)	76.0 (±4.9)	66.5 (±6.5)

Total mean (±SD) beam-flow angles: without slanted gel pads: 66.7° (±4.2), with slanted gel pads: 56.1° (±5.8), P value: 0.001; Total mean (±SD) PSVs: without slanted gel pads: 92.0 (±17.4) cm/s, with slanted gel pads: 76.9 (±10.9) cm/s, P value: 0.001

## Discussion

This study shows that beam-flow angles and PSVs decrease significantly when using slanted gel pads for carotid artery flow measurements by decreasing spectral broadening and associated overestimation of PSV (Winkler and Wu 1995). Also, as the coefficients of variation decrease with the use of gel pads, there is a potentially increased reliability of measurements.

It is often mentioned that Doppler beam-flow angles should be kept under 60°, but previous studies show that there is no single cutoff beam-flow value such as the frequently quoted less than 60° (Park et al. 2012; Allan et al. 2006; Rumack et al. 2005; Hoskins 1996; Tola and Yurdakul 2006). A prior study shows that even with Doppler angle correction, the overestimation of PSV is as high as 40 % when using a 60° angle (Winkler and Wu 1995). Another study showed even with Doppler angle correction, when comparing the average velocity measurements using 40°, 50°, 60°, 70°, 80°, and 88° beam-flow angles with 30° measurements, there was an average 8, 22, 36, 80, 160, and 1040 % increase in velocity measurements, respectively (Park et al. 2012). Therefore, decreasing the beam-flow angle is necessary to minimize flow overestimation and obtain the most accurate velocity measurements. A prior study mentioned that the average variation of intraprobe and intermachine differences for measurement at less than or equal to 70° beam-flow angles is less than or equal to 5 % and that intermachine variations in measurements are less important than using consistent angles. However, this is not the case when beam-flow angles are greater than 70° as intermachine variations were increased to 54 % probably due to error by cosine term in Doppler equation (Park et al. 2012; Tola and Yurdakul 2006). Therefore when measuring velocity in vessels with potential large beam-flow angles, it is necessary to keep not only a consistent beam-flow angle but also decrease the beam-flow angle for more reliable measurements.

The focus of this study is on the potential use of gel pads for decreasing beam-flow angles for velocity measurements in vessels which are flowing nearly parallel to the body surface such as the carotid arteries, portal veins, and femoral arteries. Although a flow phantom may have provided the true velocity of each measured value, we decided on using a human volunteer rather than a flow phantom since flow phantoms do not display the elastic properties of skin and soft tissue which alter the range of beam-flow angles that can be measured (Fig. 2b). This is why that although that even though an 18° slanted gel pad was used, the difference in mean beam-flow angle and gel pad is less than 18° (Table 1). A prior study demonstrated that Doppler velocity measurements made at different insonation angles show considerable differences which is probably due to the above mentioned spectral broadening and overestimation (Tola and Yurdakul 2006). Their study showed that velocity measurements increased as beam-flow angles increase which is consistent with the results of our study, while also mentioning that angle-specific thresholds must be determined.

Although slanted gel pads are helpful in decreasing beam-flow angles and overestimation, it will still be necessary in most cases for angle-specific thresholds to be made available. Future research into the use of multiple slanted gel pads with varied angles may be helpful in allowing measurements of fixed beam-flow angle measurements. Such gel pads would allow an operator to more easily obtain not only smaller beam-flow angles, but a specific beam-flow angle. A prior study considered 45° as the smallest beam-flow angle commonly applicable in carotid artery velocity measurements, while noting that 3 % of internal carotid arteries and 7 % of common carotid arteries could not be measured as 45° (Tola and Yurdakul 2006). In our experience, the applicable beam-flow angle is much more increased in many patients and also in less experienced radiologists. Due to the elasticity of skin and soft tissue, it may be more difficult to obtain smaller beam-flow angles in thinner patients as it can be more difficult to tilt the probe (Fig. 2b). The patient in our study had a body mass index of about 21.3 which is within normal range. The thin nature of the patient and parallel orientation of carotid arteries to neck surface made it difficult to measure Doppler flow using low beam-flow angles. Of the 13 radiologists who made measurements in this study, 11 of the radiologists used average beam-flow angles between 63.6° and 73.6°, while 2 of the radiologists who designed this study used average beam-flow angles of 61.2° and 64° without the use of gel pads. This difference may be due to the two radiologists having a heightened attention and experience in decreasing beam-flow angles during measurement.

Slanted gel pads may allow decreased beam-flow angles measurements in patients where small beam-flow angles are not obtainable. Therefore, slanted gel pads have the clinical potential of allowing more accurate and reliable velocity measurements. For example, in the carotid arteries this would allow more accurate and reliable assessment of significant carotid artery stenosis. Our recommendation for clinical practice would currently be to use the smallest possible beam-flow angle for accuracy and obtain a similar beam-flow angle for follow up studies to the increase reliability of velocity measurement comparisons.

Limitations of our method include the potential need for angle-specific thresholds to be made available in the literature. If beam-flow angles are decreased by a large amount, the thresholds used in the literature will probably be too high. This may be overcome by research into angle-specific thresholds, mathematical conversion of velocity overestimation by beam-flow angles, and the use of varied angled slanted gel pads. It should be noted that the slanted gel pad can slide off during measurements and we had the volunteer sometimes hold the gel pad into place, but this may be overcome by manufacture of slanted gel pads with specific shapes preventing such inconveniences. Limitations of our study include the inclusion of only one volunteer without real testing in clinical situations, but as previously mentioned our study used a single volunteer rather than a flow phantom because commercial flow phantoms do not display the elastic properties of skin and soft tissue, but we believe that our method is adequate for the purpose of our study. Due to commercial gel pads only being available to us in 2 cm thickness, only slanted gel pads with a fixed 18° were created and used in our study. Thicker and more angled gel pads will allow more decreased beam flow angles. Another potential pitfall is if the Doppler angle is too low, spectral broadening may also occur due to the finite dimensions of the range gate, but we do not feel this limitation nearly as significant as increased Doppler angles (Yu et al. 2006). Separate further research into angle specific thresholds for various clinical situations, clinical use of slanted gel pads, and the use of multiple varied angled slanted gel pads appears warranted.

## Conclusions

The use of slanted gel pads decrease beam-flow angles and decrease overestimation of Doppler flow velocity measurements while potentially increasing the reliability of measurements.

## Abbreviations

PSV: peak systolic flow velocity
SD: standard deviation

## References

[CR1] Allan PL, Dubbins PA, Pozniak MA, McDicken WN (2006). Clinical Doppler ultrasound.

[CR2] Heijenbrok-Kal MH, Buskens E, Nederkoorn PJ, van der Graaf Y, Hunink MG (2006). Optimal peak systolic velocity threshold at duplex us for determining the need for carotid endarterectomy: a decision analytic approach. Radiology.

[CR3] Hoskins PR (1996). Accuracy of maximum velocity estimates made using Doppler ultrasound systems. Br J Radiol.

[CR4] Hoskins PR (1999). A comparison of single- and dual-beam methods for maximum velocity estimation. Ultrasound Med Biol.

[CR5] Park MY, Lee YJ, Rha SE, Oh SN, Byun JY, Kim DG (2008). Correlation of portal venous velocity and portal venous flow with short-term graft regeneration in recipients of living donor liver transplants. Transplant Proc.

[CR6] Park MY, Jung SE, Byun JY, Kim JH, Joo GE (2012). Effect of beam-flow angle on velocity measurements in modern Doppler ultrasound systems. AJR Am J Roentgenol.

[CR7] Rumack CM, Wilson SR, J WC, Levine D (2005). Diagnostic ultrasound.

[CR8] Tola M, Yurdakul M (2006). Effect of Doppler angle in diagnosis of internal carotid artery stenosis. J Ultrasound Med.

[CR9] Winkler AJ, Wu J (1995). Correction of intrinsic spectral broadening errors in Doppler peak velocity measurements made with phased sector and linear array transducers. Ultrasound Med Biol.

[CR10] Yu AC, Steinman AH, Cobbold RS (2006). Transit-time broadening in pulsed Doppler ultrasound: a generalized amplitude modulation model. IEEE Trans Ultrason Ferroelectr Freq Control.

